# FoxG1 Directly Represses Dentate Granule Cell Fate During Forebrain Development

**DOI:** 10.3389/fncel.2018.00452

**Published:** 2018-11-23

**Authors:** Xiao Han, Xiaochun Gu, Qianqian Zhang, Qingxia Wang, Yao Cheng, Samuel J. Pleasure, Chunjie Zhao

**Affiliations:** ^1^Key Laboratory of Developmental Genes and Human Diseases, Ministry of Education, School of Medicine, Southeast University, Nanjing, China; ^2^Programs in Neuroscience and Developmental Stem Cell Biology, Department of Neurology, Weill Institute for Neuroscience, University of California, San Francisco, San Francisco, CA, United States

**Keywords:** *Foxg1*, cell fate determination, neurogenesis, granule cell, Cajal-Retzius cells

## Abstract

The cortex consists of 100s of neuronal subtypes that are organized into distinct functional regions; however, the mechanisms underlying cell fate determination remain unclear. *Foxg1* is involved in several developmental processes, including telencephalic patterning, cell proliferation and cell fate determination. Constitutive disruption of *Foxg1* leads to the transformation of cortical neurons into Cajal-Retzius (CR) cells, accompanied by a substantial expansion of the cortical hem through the consumption of the cortex. However, rather than the induction of a cell fate switch, another group has reported a large lateral to medial repatterning of the developing telencephalon as the explanation for this change in cell type output. Here, we conditionally disrupted *Foxg1* in telencephalic progenitor cells by crossing *Foxg1^fl/fl^* mice with *Nestin-CreER^TM^* mice combined with tamoxifen (TM) induction at distinct developmental stages beginning at E10.5 to further elucidate the role of FoxG1 in cell fate determination after telencephalon pattern formation. The number of dentate gyrus (DG) granule-like cells was significantly increased in the cortex. The increase was even detected after deletion at E14.5. *In vivo* mosaic deletion and *in vitro* cell culture further revealed a cell-autonomous role for FoxG1 in repressing granule cell fate. However, the cortical hem, which is required for the patterning and the development of the hippocampus, was only slightly enlarged and thus may not contribute to the cell fate switch. Lef1 expression was significantly upregulated in the lateral, cortical VZ and FoxG1 may function upstream of Wnt signaling. Our results provide new insights into the functions of FoxG1 and the mechanisms of cell fate determination during telencephalic development.

## Introduction

The large variety of neuronal cell types in the cerebral cortex arises from the neuroepithelium. During early development, beginning with regionalization, specific cell types are produced under the control of both extrinsic morphogens secreted by signaling centers and cell-intrinsic transcription factors expressed in gradients along coordinate axes (Bishop et al., 2002; Fukuchi-Shimogori and Grove, 2003; Hamasaki et al., 2004; Shimogori et al., 2004; Toyoda et al., 2010; Alfano et al., 2011). Cajal-Retzius (CR) cells are among the earliest born pioneer neurons and are mainly produced in the time window from E10.5 to E14.5 (Gorski et al., 2002; Yamazaki et al., 2004; Bielle et al., 2005). Cortical neurons are born in a sequential order from E12.5-E17.5, with deep layer neurons produced first followed by upper layer neurons (Mcconnell and Kaznowski, 1991; Caviness and Takahashi, 1995; Tan et al., 1998; Shen et al., 2006; Molyneaux et al., 2007). Hippocampal cells such as dentate gyrus (DG) granule cells are generated from the medial pallium and their birth is initiated at E13 and continues at high levels until postnatal day 15 in mice (Machon et al., 2007; Yu et al., 2014) before receding to a lower ongoing adult level. According to Deguchi et al. (2011) neurogenesis begins in the developing DG as early as E10. However, researchers have not clearly elucidated the mechanism responsible for the spatiotemporal determination of these specific cell fates.

The cortical hem produces signaling molecules, for example Wnts, which have previously been shown to play critical roles in pattern formation and hippocampal development (Grove et al., 1998; Galceran et al., 2000; Lee et al., 2000; Shimogori et al., 2004; Machon et al., 2007). A deficiency in *Wnt3a*, a ligand that is specifically expressed in the hem, leads to the loss of the hippocampus (Lee et al., 2000). Meanwhile, overexpression of Wnt signaling molecules in the dorsal pallium results in the ectopic generation of Prox1^+^ DG granule cells in the dorsal cortex (Machon et al., 2007). *Lef1*, a downstream effector of the Wnt signaling pathway, has been reported to be crucial for the development of the DG (Galceran et al., 2000). Although many researchers have attempted to explain these findings, the mechanisms that control the specification of cell fate require further exploration.

*Foxg1* has been shown to be a key regulator of telencephalic cell fate determination. As shown in the study by Hanashima et al. (2004, 2007) constitutive disruption of *Foxg1* leads to the expansion of the cortical hem by consuming the neocortex, and almost all cortical neurons switch their fates to CR cells. Based on the results from studies by Muzio and Mallamaci (2005), the overproduction of CR cells results from large-scale lateral-to-medial repatterning. In the present study, the *Nestin-CreER^TM^* line was employed to conditionally ablate *Foxg1* at E10.5, E12.5 and E14.5 and to further investigate the function of FoxG1 in cell fate determination after the pattern of the telencephalon formed. Interestingly, a large proportion of cortical *Foxg1*-deficient cells switched their fates to DG granule-like cells. Studies of the mosaic deletion of *Foxg1* and cell culture *in vitro* revealed a cell-autonomous role of FoxG1. Our results provide new insights into the functions of FoxG1 in cell fate determination.

## Materials and Methods

### Animals

*Foxg1^fl/fl^* mice were generated as previously reported (Tian et al., 2012). The *Nestin-CreER^TM^* and *ROSA26-YFP* reporter mice were purchased from The Jackson Laboratory. For *Foxg1* conditional disruption in neural progenitor cells, *Nestin-CreER^TM^* mice were crossed with *Foxg1^fl/fl^* mice and induced with tamoxifen. *ROSA26-YFP* mice were employed for cell tracing. The day on which the plug was detected was designated E0.5. All animals were bred in the animal facility at Southeast University. All experiments were performed according to guidelines approved by Southeast University.

### Tamoxifen Induction and Tissue Preparation

Tamoxifen (Sigma-Aldrich, T5648–5G) was dissolved in corn oil (Sigma-Aldrich, C8267) at a concentration of 20 mg/ml. For typical induction protocols, tamoxifen (TM) was intraperitoneally injected into pregnant mice at a concentration of 4 mg/40 g body weight. For mosaic deletion, a low dosage of 2–3 mg/40 g body weight or a very low dosage of 1–2 mg/40 g body weight was used. For tissue preparation, E12.5-E14.5 brains were dissected from embryos in cold 0.1 M PBS and then immersed in 4% paraformaldehyde (PFA) overnight at 4°C. For the collection of E18.5 brains, embryos were first perfused intracardially with 4% PFA, and brains were then dissected and post-fixed for 8–12 h at 4°C. After cryoprotection with 30% sucrose, brains were embedded in OCT. Twelve-micrometer-thick coronal sections were obtained using a Leica cryostat (CM 3050S) and stored at -70°C until use.

### Immunofluorescence Staining

The immunostaining of tissue sections was performed as previously described (Tian et al., 2012). For staining of cultured cells, culture slides were first rinsed with PBS, fixed with 4% PFA for 15 min, and then blocked with 10% calf serum in PBS containing 0.1% Triton X-100 (PBT) for 30 min. Slides were then incubated with primary antibodies diluted in 10% calf serum overnight at 4°C. Subsequently, slides were washed with PBT 5 times, and incubated with the secondary antibodies for 4 h at room temperature. Finally, slides were cover-slipped after washes with PBS. The following antibodies and reagents were used in the present study: chicken anti-GFP (Abcam, AB13970, 1:1000); goat anti-CalR (Millipore, AB1550, 1:500); goat anti-Prox1 (R&D, AF2727, 1:250); mouse anti-Reelin (Millipore, MAB5364, 1:1000); mouse anti-Satb2 (Santa Cruz, SC81376, 1:500); rabbit anti-CalR (Millipore, AB5054, 1:1000); rabbit anti-FoxG1 (Abcam, AB18259, 1:250); rabbit anti-Foxp2 (Abcam, AB16046, 1:1000); rabbit anti-Lhx2 (Abcam, AB184337, 1:500); rabbit anti-Pax6 (Covance, 1:1000); rabbit anti-Tbr1 (Millipore, AB10554, 1:500); rat anti-Ctip2 (Abcam, AB18465, 1:1000); Alexa Fluor 488-conjugated goat anti-chicken (Invitrogen, A11039, 1:500); DyLight 488-conjugated donkey anti-rabbit (Thermo Fisher Scientific, SA5-10038, 1:500); DyLight 650-conjugated donkey anti-rabbit (Thermo Fisher Scientific, SA5-10041, 1:500); DyLight 488-conjugated donkey anti-goat (Thermo Fisher Scientific, SA5-10086, 1:500); Alexa Fluor 546-conjugated rabbit anti-goat (Invitrogen, A21085, 1:500); DyLight 488-conjugated donkey anti-mouse (Thermo Fisher Scientific, SA5-10066, 1:500); DyLight 550-conjugated donkey anti-mouse (Thermo Fisher Scientific, SA5-10067, 1:500); Alexa Fluor 488-conjugated goat anti-rat (Invitrogen, A11006, 1:500); Alexa Fluor 546-conjugated goat anti-rat (Invitrogen, A11081, 1:500); and DAPI (Sigma-Aldrich, D9564, 1:1000).

### *In situ* Hybridization

*In situ* hybridization was performed as previously described (Yang et al., 2009). Briefly, tissues were fixed in 4% PFA overnight, cryoprotected in 30% sucrose/DEPC-PBS at 4°C and then embedded in OCT. Coronal sections (thickness: E12.5: 8 μm, E14.5: 10 μm, E18.5: 12 μm) were obtained using a Leica cryostat (CM 3050S) and stored at 70°C until use. The probes were amplified using the following primers: *Lef1*: Forward: 5′-GGAAAACCGAAGCGAAAGGG-3′ and Reverse: 5′-AGTTGGAAGACTGAGTGCGG-3′. The *Wnt2b* plasmid was a kind gift from *Thomas Theil* (University of Edinburgh, Edinburgh, United Kingdom). The *Wnt3a* (426103) and *Ephb1* (6821724) cDNAs were purchased from *the QriGene.*

### Quantitative Real-Time PCR

The dorsal telencephalon was dissected from E14.5 embryos and total RNA was isolated using an RNeasy Plus Mini Kit (Qiagen, 74134). RNA was then reverse transcribed into cDNAs using the PrimeScript^TM^ RT reagent kit with gDNA Eraser (Takara, RR047A). Quantitative PCR was performed using a StepOnePlus Real-Time PCR System (Applied Biosystems) with SYBR Green fluorescent master mix (Roche, 4913914001). Q-PCR was conducted according to standard methods described in a previous study (Tian et al., 2012). Primers used for Q-PCR are listed below: *Wnt3a*: Forward: 5′-AGGTAAGCTACTCCCTCAACTA-3′, Reverse: 5′-CTGAAGCACCCTCTCATGTATC-3′; *Wnt2b*: Forward: 5′-CCTTCCTCTACCCTCAATCCT-3′, Reverse: 5′-CACTCAGCCTCCTAAATCCATC-3′; and *Lef1*: Forward: 5′-AGAACACCCTGATGAAGGAAAG-3′, Reverse: 5′-GTACGGGTCGCTGTTCATATT-3′.

### Cell Culture

For the culture of neural progenitor cells, TM induction was performed at E12 and cells were then isolated from E13.5 dorsal cerebral cortices and cultured using previously reported methods (Shen et al., 2006; Zhang et al., 2016). Briefly, cells were first cultured in proliferation-stimulating DMEM/F12 (Gibco, 11330-032) containing 20 ng/ml bFGF2 (Gibco, PMG0035), 20 ng/ml EGF (Gibco, PMG8041) and 2% B27 supplement minus vitamin A (Gibco, 12587010). Two or 3 days later, neurospheres were dissociated and transferred to differentiation-promoting DMEM/F12 (Gibco, 11330-032) containing 0.5% fetal bovine serum (FBS, Gibco, 10091148) and 2% B27 serum-free supplement (Gibco, 17504044). Cells were plated on slides at a density of 10^4^ cells per cm^2^ and cultured for 4 days before processing for immunofluorescence staining.

### Cell Counting and Data Analysis

For tissues, two 12 μm-thick coronal sections from a similar level of each E18.5 brain were selected. Cells were counted in an average value by experimenters blind to the animal genotypes. Images of immunofluorescence staining were acquired using a FluoView FV1000 confocal microscope (Olympus) with a 20× objective lens. The areas of each section that were counted were 315 μm (width) × 630 μm (height). For cultured cells, images of immunofluorescence staining were acquired with the 40× objective lens. Blinded cell counts in five or six views were selected from each plate. At least 3 pairs of brains from 3 different litters were employed for each experiment. Student’s *t*-test was used to analyze the statistical significance of differences (^∗^*P* < 0.05, ^∗∗^*P* < 0.01, ^∗∗∗^*P* < 0.001), and all values are presented as means ± SEM.

## Results

### Temporal Loss-of-Function of *Foxg1* Beginning at E10.5 Leads to the Ectopic Production of CalR^+^ Cells

**FIGURE 1:**
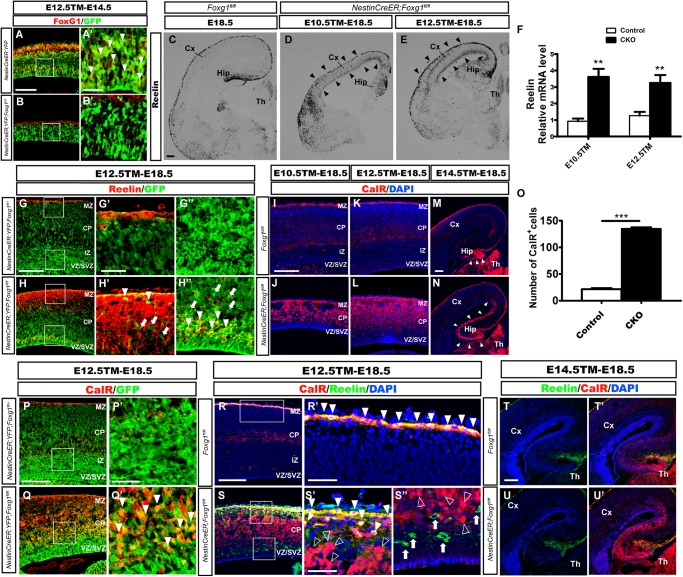
Conditional deletion of Foxg1 beginning at E10.5 led to the ectopic production of CalR^+^ cells and Cajal-Retzius cells. **(A–B’)** Immunofluorescence staining showed the successful deletion of FoxG1 in GFP^+^ cells in the *Nestin-CreER^TM^;ROSA26-YFP;Foxg1^fl/fl^* cortex when TM was administered at E12.5 and tissues were analyzed at E14.5. In controls, a large proportion of GFP^+^ cells co-labeled with FoxG1 (**A’**, arrowheads). **(A’,B’)** Show high magnification images of the boxed regions in **(A,B)**, respectively. Compared with the controls **(C)**, *in situ* hybridization for Reelin showed an obviously increased number of Reelin^+^ cells in mutants that were administered TM at E10.5 or E12.5 (**D,E**, arrowheads). **(F)** Q-PCR to detect levels of the Reelin mRNA (E10.5: *n* = 3 animals per genotype, ^∗∗^*p* = 0.0064; E12.5: *n* = 4 for Control, *n* = 3 for CKO, ^∗∗^*p* = 0.0091). **(G–H”)** Immunofluorescence staining showed a portion of GFP^+^ cells co-labeled with Reelin that were ectopically located in the dorsal-lateral plate in mutants (**H–H”**, arrowheads) compared with the controls **(G–G”)**. **(G’,H’)** show high magnification images of the boxed regions in the MZ presented in **(G,H)**; **(G”,H”)** show high magnification images of boxed regions in the VZ/SVZ presented in **(G,H)**. **(I–L)** Immunofluorescence staining showed overproduced CalR^+^ cells in the cortical plate in mutants. **(J–L)** After *Foxg1* inactivation at E10.5 or E12.5 and an analysis of the brain tissues at E18.5, CalR was mainly expressed in the MZ and TCA in controls **(I,K)**. **(M,N)** When *Foxg1* was deleted at E14.5, a stream-like CalR^+^ cell band extending from the DG to the lateral cortex was detected in mutants (**N**, arrowheads), while CalR staining was confined to the DG in the controls (**M**, arrowheads). **(O)** The number of CalR^+^ cells was significantly increased in mutants compared with the controls after *Foxg1* disruption at E12.5 and analysis at E18.5 (*n* = 4 animals per genotype, Control: 21.5 ± 2.131; CKO: 135.4 ± 2.437, ^∗∗∗^*p* < 0.0001). **(P–Q’)** Cell tracing using YFP showed that a large proportion of *Foxg1*-ablated cells co-expressed CalR (**Q’**, arrowheads). **(P’,Q’)** Show high magnification images of the boxed regions in **(P,Q)**, respectively. **(R–S”)** Immunostaining showing a lack of Reelin co-expression in most CalR^+^ cells in mutants. **(R’,S’)** Show high magnification images of the boxed MZ regions presented in **(R,S)**, respectively. Co-localization of CalR with Reelin was observed in the MZ (solid arrowheads), while most CalR^+^ neurons positioned in the lower region of the cortical plate were Reelin^-^ (**S’**, hollow arrowheads). **(S”)** The high magnification image of the boxed VZ/SVZ region in S showed that ectopic Reelin^+^ cells were not CalR^+^ (arrows); hollow arrowheads indicated CalR^+^Reelin^-^ cells. **(T–U’)** Immunostaining showed that the ectopic CalR^+^ stream was not Reelin^+^ after *Foxg1* disruption at E14.5. CKO, conditional knockout; Cx, cortex; Hip, hippocampus; Th, thalamus; MZ, marginal zone; CP, cortical plate; IZ, intermediate zone; VZ, ventricular zone; SVZ, subventricular zone. Scale bars: **(A’–B’,G’–H”,P’–Q’,R’–S”)**: 50 μm; **(A–B,C–E,G–H,I–N,P–Q,R–S,T–U’)**: 200 μm.

*Foxg1* was deleted in neuronal progenitors at distinct developmental stages by crossing *Foxg1^fl/fl^* mice with the *Nestin*-*CreER^TM^* line (Imayoshi et al., 2006) combined with tamoxifen (TM) induction to identify the role of FoxG1 in cell fate determination beginning at E10.5. First, we detected the efficiency of *Foxg1* deletion at E14.5 after TM induction at E12.5. *Rosa26-YFP* was employed to trace *Foxg1*-ablated cells (Srinivas et al., 2001). As shown in Figures 1A–B’, FoxG1 was efficiently ablated in GFP^+^ cells in the *Nestin-CreER^TM^;*ROSA26-YFP*;Foxg1^fl/fl^* mutant cortex, compared to its strong expression in the *Nestin-CreER^TM^;ROSA26-YFP* controls. We then examined the number of CR cells by performing *in situ* hybridization for *Reelin*. As shown in Figure 1D, compared to the controls (Figure 1C), when TM was administered at E10.5, the number of Reelin^+^ cells was obviously increased in the mutant cortex (Figure 1D, arrowheads). A similar result was obtained when TM was administered at E12.5 (Figure 1E, arrowheads), consistent with previous reports (Hanashima et al., 2004, 2007). Q-PCR showed a 3-4-fold increase in Reelin expression (Figure 1F). However, a large number of Reelin^-^ cells was distributed in the cortex. Double immunostaining for Reelin and GFP was then performed in *Nestin-creER^TM^;ROSA26-YFP;Foxg1^fl/fl^* brains at E18.5. In controls, there no Reelin^+^ cells co-expressed with GFP in the cortex (Figures 1G–G”), and in mutants, only a portion of GFP^+^ cells co-expressed Reelin (Figures 1H–H”, arrowheads), whereas many GFP^+^*Foxg1-*deficient cells were Reelin^-^ (Figures 1H–H”, arrows), indicating that the *Foxg1* deficiency beginning at E10.5 caused a partial but not total conversion of cortical neurons to CR fates.

We performed immunostaining for Calretinin (CalR), another marker of CR cells (Hevner et al., 2003; Bielle et al., 2005), to further confirm this observation. After TM administration at E10.5 or E12.5, strong CalR expression was detected in CR cells located in the MZ and TCA in E18.5 control brains, as well as sparsely distributed interneurons in the cortex (Figures 1I,K). However, in mutants, a large proportion of CalR^+^ cells occupied the whole cortical plate (Figures 1J,L). Noticeably, the staining pattern was very different from Reelin (Figures 1D,E), and a much greater number of CalR^+^ cells was detected than Reelin^+^ cells (Figures 1J,L). When *Foxg1* was deleted at E14.5, an increase in the number of CalR^+^ cells was noted in the area of medial wall, and a thick CalR^+^ cell band extended from the DG to the lateral cortex. In the controls, CalR staining was only limited to the DG (Figures 1M,N, arrowheads). As shown in Figure 1O, the number of CalR^+^ cells was increased more than 6-folds when a 315 μm width, 630 μm height region was quantified in the lateral putative cortex after *Foxg1* ablation at E12.5. We also performed double labeling for CalR with GFP and found that many CalR^+^ cells were GFP^+^ (Figures 1P–Q’, arrowheads), suggesting that these cells were derivatives of *Foxg1-*deficient progenitor cells.

Double immunostaining was performed after *Foxg1* deletion at E12.5 to determine whether these CalR^+^ cells co-expressed Reelin. As shown in Figures 1R–S”, the co-localization of CalR with Reelin at E18.5 was mainly observed in the MZ (Figures 1S–S”, solid arrowheads), while the majority of CalR^+^ cells in the cortical plate were Reelin^-^ (Figures 1S–S”, hollow arrowheads), strongly indicating that most CalR^+^ cells were not CR cells. When TM induction was performed at E14.5, the ectopically produced CalR^+^ cells did not co-express Reelin (Figures 1T–U’). Taken together, the conditional disruption of *Foxg1* beginning at E10.5 led to the ectopic production of CalR^+^ cells, and these CalR^+^ cells were not CR cells. Based on our data, FoxG1 may play a distinct role during cortical cell fate determination.

### Most *Foxg1*-Deficient CalR^+^ Cells Are DG Granule-Like Cells Rather Than Cortical Neurons

**FIGURE 2:**
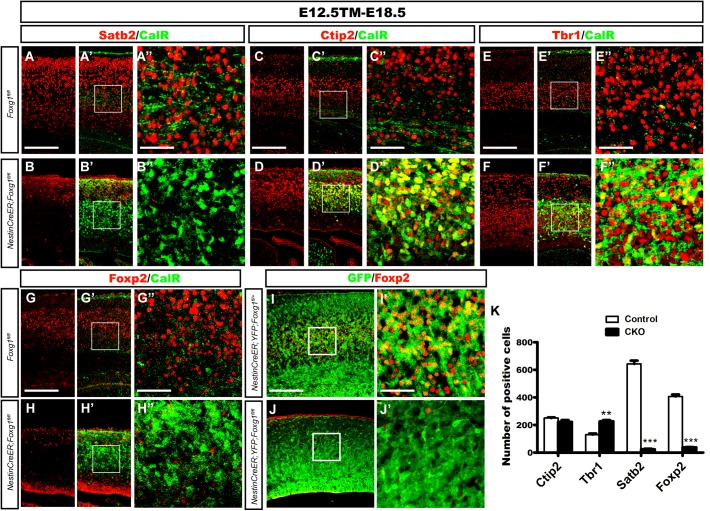
Overproduced CalR^+^ cells were not cortical excitatory neurons. **(A–B”)** Immunostaining showed that CalR^+^ cells were Satb2^-^ after *Foxg1* was deleted at E12.5 and brains were analyzed at E18.5. **(A”,B”)** Show high magnification images of the boxed regions in **(A’,B’)**, respectively. **(C–F”)** Double staining of CalR with Ctip2 or Tbr1 showed that, compared with the controls **(C–C”,E–E”)**, a large proportion of CalR^+^ cells located in the cortical plate co-expressed Ctip2 and Tbr1 **(D–D”,F–F”)**. **(C”,D”,E”,F”)** High magnification images of the boxed regions in **(C’,D’,E’,F’)** respectively. **(G–H”)** No co-localization of Foxp2 with CalR was detected in the mutant cortical plate after *Foxg1* inactivation at E12.5. **(G”,H”)** Show high magnification images of the boxed regions in **(G’,H’)**, respectively. **(I–J’)** *Foxg1*-ablated cells labeled with YFP rarely co-expressed Foxp2 in the mutants **(J,J’)** compared with the controls **(I,I’)**. **(I’,J’)** Show high magnification images of the boxed regions in **(I”,J”)**, respectively. **(K)** Statistical analysis of Satb2^+^ and Foxp2^+^ cells in the dorsal-lateral cortex (Ctip2, *n* = 3 mice per genotype, Control: 250.7 ± 4.343; CKO: 222.3 ± 13.05, ns, *p* = 0.1085; Tbr1, *n* = 3 mice per genotype, Control: 130.5 ± 7.522; CKO: 226.0 ± 9.504, ^∗∗^*p* = 0.0014; Satb2, *n* = 3 mice per genotype, Control: 642.7 ± 23.69; CKO: 23.67 ± 4.419, ^∗∗∗^*p* < 0.0001; and Foxp2: *n* = 4 mice per genotype, Control: 407.0 ± 14.0; CKO: 37.63 ± 2.726, ^∗∗∗^*p* < 0.0001). Scale bars: **(A”,B”,C”,D”,E”,F”,G”,H”,I’,J’)**: 50 μm; **(A–B’,C–D’,E–F’,G–H”,I,J)**: 200 μm.

We performed double immunostaining for CalR and markers specific for callosal or corticofugal deeper layer cortical neurons to determine whether the CalR^+^ cells in the cortex might be cortical excitatory neurons. As shown in Figures 2A–B”, when TM induction was performed at E12.5 and brains were examined at E18.5, a large number of Satb2^+^ callosal projection neurons were distributed in the cortical plate of control brains (Figures 2A–A”), while very few Satb2^+^ cells were observed in mutants. No co-localization of CalR and Satb2 was detected (Figures 2B–B”,K). We then examined Ctip2 or Tbr1, markers for corticofugal Layer 5 and 6 neurons, respectively (Hevner et al., 2001; Arlotta et al., 2005). As shown in Figures 2C–F”, a large population of CalR^+^ cells co-expressed Ctip2 or Tbr1 in the mutants. Since both Ctip2 and Tbr1 are also expressed in DG granule cells (Hodge et al., 2008; Roybon et al., 2009; Simon et al., 2012), these neurons might adopt either a DG-like or Layer5/6 fate. Next, Foxp2, a marker that is only expressed in cortical deep layer neurons (Ferland et al., 2003), was assessed. As shown in Figures 2G–H”, many Foxp2^+^ neurons were not CalR^+^ in controls. However, remarkably, in mutants, Foxp2^+^ neurons had almost completely disappeared (Figures 2H–H”), indicating that CalR^+^ cells were not deeper layer neurons. This result was further confirmed by cell tracing using *ROSA26-YFP* mice. Compared with many GFP^+^ cells that co-expressed Foxp2 in the *Nestin-CreER^TM^;ROSA26-YFP;Foxg1^fl/+^* control, none of the GFP^+^ cells co-expressed Foxp2 in the mutants (Figures 2I–J’). The statistical analysis revealed a substantial decrease in the number of Foxp2^+^ neurons accompanied by an increase in the number of Tbr1^+^ cells and no obvious change in the number of Ctip2^+^ cells when a 315 μm width, 630 μm height region was quantified in the lateral putative cortex (Figure 2K). Thus the *Foxg1* deficiency alters cortical neuron fates.

**FIGURE 3:**
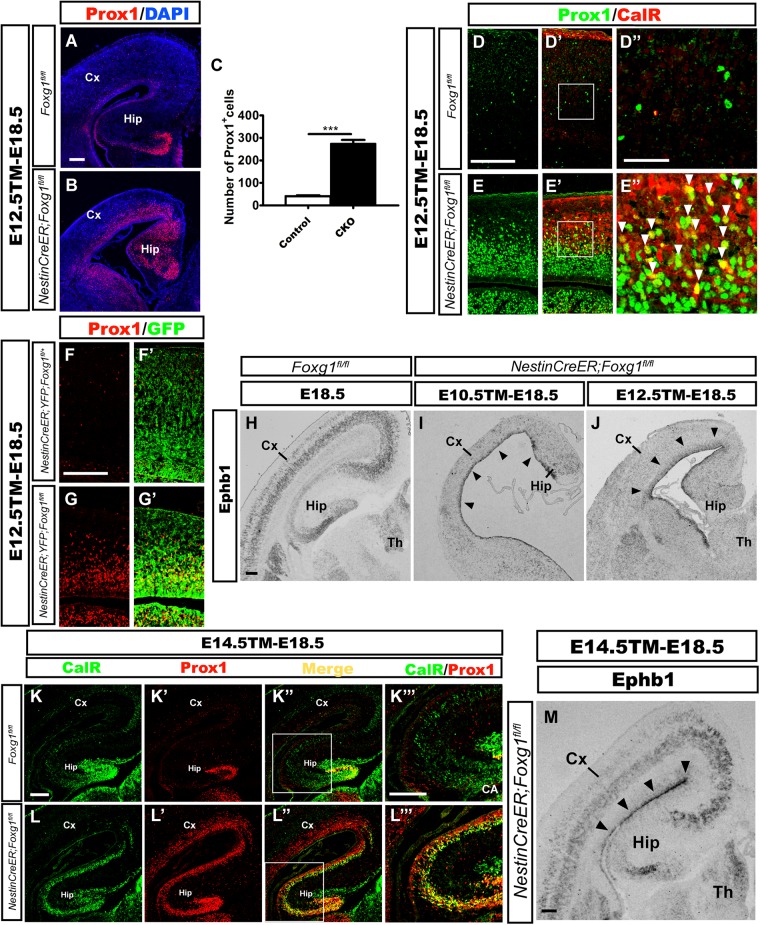
Overproduced CalR^+^ cells acquired a DG granule-like cell fate. **(A,B)** Immunostaining showing increased Prox1^+^ cells in the mutant cortical plate **(B)** after *Foxg1* inactivation at E12.5, while Prox1^+^ cells were limited to the DG in controls **(A)**. **(C)** The number of Prox1^+^ cells was significantly increased in mutants compared with the controls (*n* = 3 mice per genotype, Control: 41.0 ± 4.163; CKO: 275.0 ± 16.04, ^∗∗∗^*p* < 0.0001). **(D–E”)** Many CalR^+^ cells in the mutant cortical plate co-expressed Prox1 (**E’,E”**, arrowheads) compared with the controls **(D’,D”)**. **(D”,E”)** Show high magnification images of the boxed regions in **(D’E’)**, respectively. **(F–G’)** *Foxg1*-ablated cells labeled with YFP co-expressed Prox1. **(H–J)** *In situ* hybridization staining for *Ephb1* after *Foxg1* ablation at E10.5 **(I)** or E12.5 **(J)**; *Ephb1* was ectopically expressed in the VZ/SVZ throughout the cortex (arrowheads), while hybridization in the controls **(H)** was limited to the developing DG and cortical plate. **(K–L”’)** The overproduced CalR^+^ stream extending from the DG to the cortex co-expressed Prox1 after *Foxg1* deletion at E14.5. **(K”’,L”’)** Show high magnification images of the boxed regions in **(K”,L”)**, respectively. **(M)** *In situ* hybridization for *Ephb1* after *Foxg1* ablation at E14.5; *Ephb1* was ectopically expressed in the VZ/SVZ throughout the cortex (arrowheads). CA: cornu ammonis area. Scale bars: **(D”,E”)**: 50 μm; **(A,B,D–E’,F–M)**: 200 μm.

According to previous studies, CalR is also expressed in immature granule cells during the development of the mouse DG (Brandt et al., 2003; Lavado et al., 2010); therefore, we then examined whether these ectopic CalR^+^ cells co-expressed Prox1, a specific molecular marker for granule cells and its progenitors (Liu et al., 2000; Jessberger et al., 2008; Lavado et al., 2010). When TM was administered at E12.5 and brains were analyzed at E18.5, a large number of Prox1^+^ cells populated the cortical plate in the mutant brains (Figure 3B). Cell counting showed an approximately 7-fold increase in the number of Prox1^+^ cells in the lateral putative cortex (Figure 3C), many of which were CalR^+^ (Figures 3E–E”). In contrast, the distribution of Prox1^+^ cells was only limited to the developing DG in controls (Figure 3A), and no obvious co-localization of CalR with Prox1 was detected in the cortical plate (Figures 3D–D”), strongly suggesting that the majority of CalR^+^ cells were immature granule-like cells. Cell tracing was performed to further examine the transformation of cell fate after *Foxg1* deletion. As shown in Figures 3F–G’, few GFP^+^ cells co-expressed Prox1in the *NestinCreER;ROSA26-YFG;Foxg1^fl/+^* control cortex, while most of the *Foxg1-*deficient GFP^+^ cells were Prox1^+^, consistent with the double immunostaining for CalR with Prox1.

*Ephb1* is expressed at high levels in DG progenitors and is critical for DG development (Chumley et al., 2007). Upregulation of *Ephb1* has been observed after constitutive *Foxg1* ablation, reflecting the expansion of the DG area (Muzio and Mallamaci, 2005; Godbole et al., 2018). Here, we detected *Ephb1* expression using *in situ* hybridization at E18.5 after *Foxg1* inactivation at E10.5 and E12.5 to further elucidate the role of FoxG1. In controls, *Ephb1* was mainly expressed in the developing DG and cortical plate (Figure 3H), while strong *Ephb1* staining was remarkably expanded from the medial VZ to the lateral cortical VZ/SVZ in mutants (Figures 3I,J, arrowheads), providing further support for cell fate transformation. Based on our results, most *Foxg1-*deficient cells switch to a DG granule-like cell fate.

The conventional deletion of *Foxg1* (which occurs at a very early age of approximately E9.0) results in lateral-to-medial repatterning of the cortical primordium (Muzio and Mallamaci, 2005). We deleted *Foxg1* at E14.5, well after the time point when the cortical patterning is complete (Shimogori et al., 2004; Storm et al., 2006; Borello and Pierani, 2010), to ensure that the observed increase in the number of cells displaying a DG-like fate was not a result of repatterning. In mutants, strong staining for both CalR and Prox1 extended from the medial developing DG to the lateral cortex (Figures 3K–L”’). *Ephb1* was expressed at high levels in an area extending from the medial VZ to the lateral VZ (Figure 3M, arrowheads). Thus, the repressive effect of FoxG1 on granule cell fate is likely a direct effect on cell fate rather than a manifestation of repatterning.

### The Cortical Hem Is Only Slightly Expanded After *Foxg1* Deletion Beginning at E10.5

**FIGURE 4:**
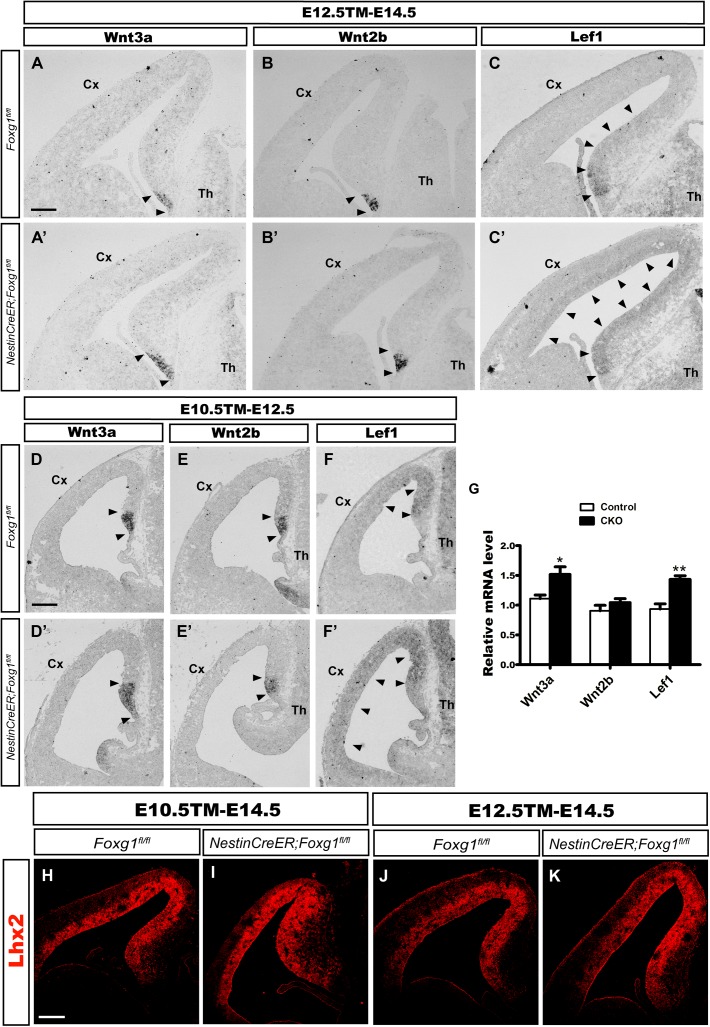
*Foxg1* inactivation at E10.5 or E12.5 led to a slight expansion of the cortical hem. The expression of Wnt3a, a specific marker of the cortical hem, was only slightly increased in the mutants (**A’,D’**, arrowheads) compared with the controls (**A,D**, arrowheads) after TM induction at E10.5 **(A,A’)** or E12.5 **(D,D’)**. No significant changes in Wnt2b expression were observed between mutants (**B’,E’**, arrowheads) and controls (**B,E**, arrowheads). **(C,C’,F,F’)** *In situ* staining showed obvious upregulation of the levels of the Lef1 transcript in the mutant medial wall and dorsal-lateral VZ (**C’,F’**, arrowheads) compared with the controls (**C,F**, arrowheads) after *Foxg1* inactivation at E12.5 or E10.5. **(G)** Q-PCR for Wnt3a, Wnt2b and Lef1 after *Foxg1* inactivation at E12.5 and an analysis of the brains at E14.5 (Wnt3a: *n* = 4 for Control, *n* = 3 for CKO, ^∗^*p* = 0.0248; Wnt2b: *n* = 4 for Control, *n* = 4 for CKO, ns *p* = 0.2719; Lef1: *n* = 4 for Control, *n* = 4 for CKO, ^∗∗^*p* = 0.0034). **(H,I,J,K)** Immunostaining for Lhx2 at E14.5, showed the expression level of Lhx2 in CKO was comparable to the control. Scale bars: 200 μm.

The cortical hem is a critical organizer of hippocampal development (Grove et al., 1998; Lee et al., 2000; Mangale et al., 2008). We examined the expression of Wnt3a and Wnt2b, well-known markers of the cortical hem (Grove et al., 1998), to further explore the consequences of timed *Foxg1* deletion on the cortical hem and the contribution of the hem to cell fate transformation. As shown in Figures 4A,A’,D,D’, the region expressing Wnt3a was only slightly enlarged, and substantial expansion to the lateral cortex was not observed. Meanwhile, Wnt2b was expressed at levels comparable to controls (Figures 4B,B’,E,E’). Thus, a significant morphological change in the hem was not observed after *Foxg1* deletion beginning at E10.5.

The Wnt signaling pathway plays important roles during hippocampal formation and DG neurogenesis (Galceran et al., 2000; Danesin et al., 2009; Caronia et al., 2010; Choe et al., 2013). We performed *in situ* hybridization for Lef1, a downstream transcription factor in the Wnt signaling pathway, to further elucidate the molecular mechanism underlying the cell fate switch. In controls, Lef1 was expressed at high levels in the DG primordium, the expression level gradually decreased along the medial wall, and finally became undetectable in the lateral cortex (Figures 4C,F). However, in mutants, Lef1 expression extended from the DG primordium to the lateral cortex and was significantly upregulated (Figures 4C’,F’). Q-PCR further confirmed the increased levels of the Wnt3a and Lef1 mRNAs. No remarkable change in Wnt2b expression was detected, consistent with the results from *in situ* hybridization (Figure 4G). Previously, it has been reported that at the time point of E9.5 *Foxg1* functions upstream of Lhx2 to control the hem formation, deletion of *Foxg1* results in an ectopic hem accompanied by the loss of Lhx2 expression, and transform its adjacent area into DG-like region (Godbole et al., 2018). Here, we examined the expression of Lhx2 at E14.5 when *Foxg1* was deleted at E10.5 and E12.5, respectively, no obvious changes were detected (Figures 4H–K), suggesting a spatiotemporal role for FoxG1 during the development of the telencephalon. Based on our data, FoxG1 may repress DG granule neuron fate by functioning upstream of Wnt signaling. The cortical hem itself did not show significant morphological alterations and might not contribute to the cell fate switch observed in this study.

### Mosaic Deletion Reveals a Cell-Autonomous Role for FoxG1 in Cell Fate Control

**FIGURE 5:**
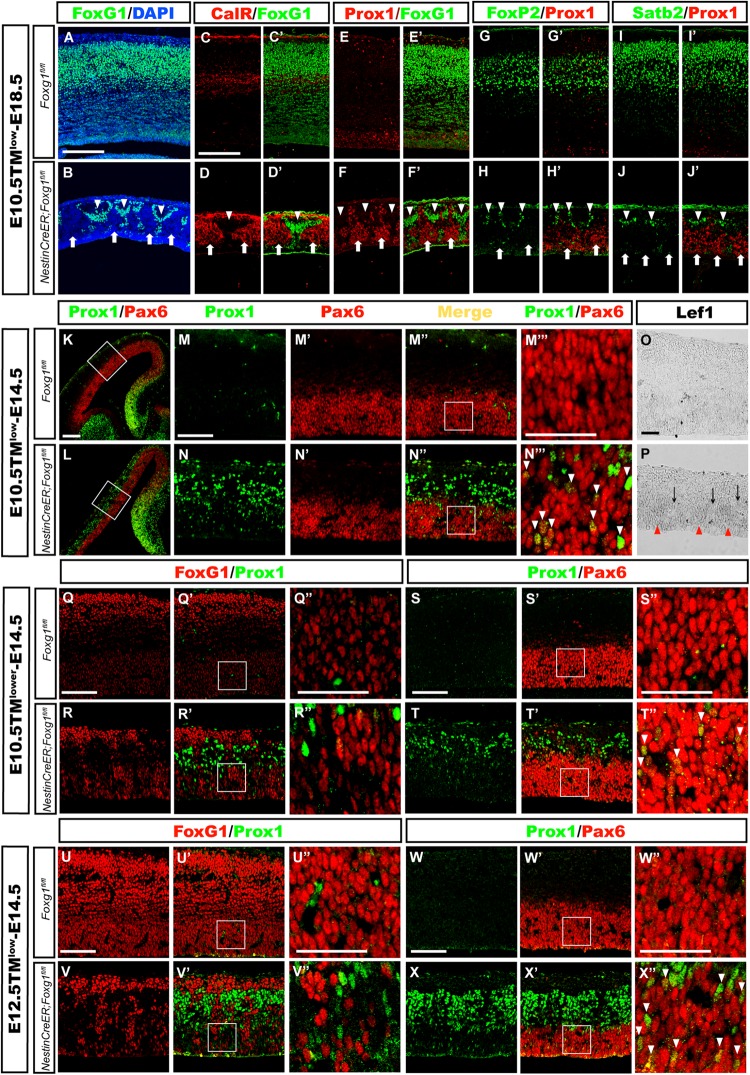
Mosaic deletion of *Foxg1* during early development revealed that FoxG1 cell-autonomously repressed the granule cell fates. **(A,B)** Mosaic deletion of *Foxg1* in the cortical plate (**B**, arrows), while the adjacent cells were FoxG1^+^ (**B**, arrowheads) after an injection of a low dose of TM at E10.5 and analysis at E18.5. **(C–D’)** CalR expression was confined to *Foxg1*-deleted cells (arrows), while adjacent FoxG1-positive cells did not co-express CalR (arrowheads).*(**(E–F’)** Only *Foxg1*-deleted cells expressed Prox1 (arrows), while adjacent FoxG1-positive cells did not co-express Prox1 (arrowheads). **(G–J’)** Normal progenitors, but not adjacent *Foxg1-*deficient cells, developed into Foxp2^+^**(G–H’)** or Satb2^+^**(I–J’)** cortical excitatory neurons. **(K,L)** Double immunostaining for Prox1 with Pax6 after the injection of a low dose of TM at E10.5 and analysis at E14.5. **(M–N”)** High magnification images of the boxed regions in **(K,L)**, respectively. In the mutants **(N–N”)**, Prox1^+^ cells exhibited a mosaic distribution pattern. **(M–N”’)***Foxg1-*deficient cells exhibited strong staining for Prox1, indicating that they were postmitotic granule cells located in the upper region of the putative cortical plate. *Foxg1-*deficient Pax6^+^ progenitors in the VZ/SVZ expressed Prox1 at low levels (**N”’**, arrowheads). **(M”’,N”’)** Show high magnification images of the boxed regions in **(M”,N”)**, respectively. **(O,P)** Mosaic deletion of *Foxg1* was accompanied by a mosaic expression pattern of *Lef1*. Red arrowheads: *Foxg1*-deleted regions; black arrows: *Foxg1*-expressing regions. **(Q–R”)** Double immunostaining showed that a few of progenitors were *Foxg1*-ablated cells that expressed Prox1 in the mutant dorsal-lateral cortex after the injection of a lower dose of TM at E10.5 and analysis at E14.5. **(Q”,R”)** Show high magnification images of the boxed regions in **(Q’,R’)**, respectively. **(S–T”)** Double immunostaining for Prox1 and Pax6 showed that *Foxg1*-ablated Prox1^+^ cells co-expressed Pax6^+^ in the dorsal-lateral cortical VZ/SVZ (**T”**, arrowheads). **(S”,T”)** Show high magnification images of the boxed regions in **(S’,T’)**, respectively. **(U–V”)** Double immunostaining for FoxG1 and Prox1 showed that *Foxg1*-ablated cells expressed Prox1 in the mutant dorsal-lateral cortex after the injection of a low dose of TM at E12.5 and analysis at E14.5. **(S”,T”)** show high magnification images of the boxed regions in **(S’,T’)**, respectively. **(W–X”)** Double immunostaining showed that mosaically generated Prox1^+^ cells co-expressed Pax6^+^ in the dorsal-lateral cortical VZ/SVZ (**X”**, arrowheads). **(W”,X”)** Show high magnification images of the boxed regions in **(W’,X’)**, respectively. Scale bars: **(M”’–N”’,O,P,Q”–R”,S”–T”,U”–V”,W”–X”)**: 50 μm; **(M–N”,Q–R’,S–T’,U–V’,W–X’)**: 100 μm; **(A–L)**: 200 μm. )*

Mosaic deletion of *Foxg1* at E10.5 or E12.5 was achieved by administering a low dosage of TM to ensure both the *Foxg1*-ablated progenitors and adjacent normal progenitors were located in approximately the same extracellular signaling environment, such as the same gradients of Wnt signals and to elucidate whether FoxG1 represses DG granule fate in a cell-autonomous manner. As shown in Figures 5A,B, when TM was administered at E10.5 and brains were examined at E18.5, FoxG1 was expressed in the whole cortex of controls, while FoxG1 was sporadically expressed in clusters in the cortical plate of mutants, indicating the successful mosaic ablation of *Foxg1.* Double immunostaining for FoxG1 with CalR or Prox1 was then performed to compare the fates of *Foxg1-*deficient cells and adjacent FoxG1^+^ cells. In controls, rarely CalR^+^ cells were positioned in the cortex and co-labeled with FoxG1 (Figures 5C–C’), while in mosaic mutants, CalR expression was confined to *Foxg1-*deficient cells (Figures 5D,D’, arrows). Prox1 staining exhibited a similar pattern (Figures 5E–F’), suggesting that *Foxg1-*deficient but not FoxG1^+^ cells switched their fate to CalR^+^/Prox1^+^ granule-like neurons. We also detected whether *Foxg1-*deficient Prox1^+^ cells co-expressed Foxp2 and Satb2, and no co-localization was observed, suggesting cortical neurons did not develop after *Foxg1* deletion (Figures 5G–J’).

We performed double immunostaining for Prox1 [expressed at high levels in mature granule cells and at low in DG progenitor cells (Lavado et al., 2010)] and Pax6, which labels both cortical and DG progenitors, to explore whether the cell fate switch occurred as early as in progenitors, namely, whether progenitors in the dorsal-lateral cortex adopted a DG progenitor fate. As shown in Figures 5K–N”’, after mosaic deletion of *Foxg1* induced by a low dosage of TM administered at E10.5, strong mosaic expression of Prox1 was detected in post-mitotic granule-like cells located in the upper cortical region, while weak expression of Prox1 was observed in Pax6^+^ progenitors in the *Foxg1-*deficient VZ area, but not its adjacent normal progenitors, indicating *Foxg1-*deficient progenitors in the dorsal-lateral cortex adopted a DG progenitor fate (Figures 5N”,N”’, arrowheads). Consistent with the progenitor fate switch, Lef1 expression was also upregulated in the cortical VZ and displayed a mosaic expression pattern (Figures 5O,P). To get more clear mosaic deletion pattern of *Foxg1*, we then administered a very low dose of TM and found that *Foxg1-*deficient progenitors were Prox1^+^ (Figures 5Q–T”). A similar progenitor fate switch was observed after the mosaic ablation of *Foxg1* at E12.5 (Figures 5U–X”). Thus, FoxG1 plays a cell-autonomous role in repressing the DG granule cell fate.

**FIGURE 6:**
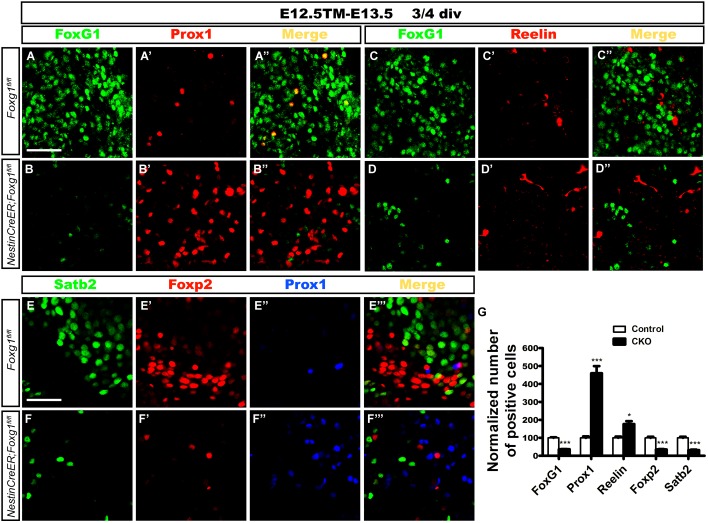
Differentiation of *Foxg1-*deficient progenitors in cell culture. **(A–B’)** Double immunostaining for FoxG1 and Prox1 in controls **(A–A”)** revealed only a few Prox1^+^ cells; however, the number of Prox1^+^ cells increased approximately 5-fold in mutants **(B–B”,G)**. **(C–D”)** Double immunostaining for FoxG1 and Reelin indicated a slight increase in the number of Reelin^+^ cells in the mutants **(D–D”)** compared with the controls **(C–C”)**. **(E–F”’)** Triple staining for Satb2, Foxp2 and Prox1. Controls exhibited many Foxp2^+^ and Satb2^+^ cortical neurons that did not co-express Prox1 **(E–E”’)**; however, the numbers of Foxp2^+^ and Satb2^+^ neurons were remarkably decreased in mutants **(F–F”’)**. **(G)** The statistical analysis of the differentiation of *Foxg1-*deficient progenitors showed increased numbers of Prox1^+^ cells and Reelin^+^ CR cells and decreased numbers of FoxG1^+^, Foxp2^+^ and Satb2^+^ cells. (FoxG1, *n* = 3 for Control; *n* = 3 for CKO, Control: 100.0 ± 3.465; CKO: 36.96 ± 0.5604, ^∗∗∗^*p* < 0.0001; Prox1, *n* = 3 for Control; *n* = 3 for CKO, Control: 100.0 ± 8.319; CKO: 459.8 ± 40.06, ^∗∗∗^*p* = 0.0009; Reelin, *n* = 3 for Control; *n* = 3 for CKO, Control: 100.0 ± 7.111; CKO: 176.7 ± 16.42, ^∗^*p* = 0.0128; Foxp2: *n* = 3 for Control, *n* = 3 for CKO, Control: 100.0 ± 6.443; CKO: 35.22 ± 1.804, ^∗∗∗^*p* = 0.0006; Satb2: *n* = 3 for Control, *n* = 3 for CKO, Control: 100.0 ± 6.111; CKO: 31.68 ± 3.294, ^∗∗∗^*p* = 0.0008). Div, days *in vitro*. Scale bars: 50 μm.

Cells were isolated from the lateral cortex at E13.5 to exclude the influence of morphogens, such as Wnts secreted from the signaling centers such as the cortical hem and to further confirm the cell-autonomous role of FoxG1. TM induction was performed at E12.5. Cells were first cultured in a proliferation medium for 2 or 3 days, and then transferred to a differential medium for 4 days to ensure that progenitors had completely differentiated into neurons, as previously described (Shen et al., 2006; Zhang et al., 2016). Double immunostaining for FoxG1 and Prox1 or Reelin was performed. As shown in Figures 6A–D”, only a few Prox1^+^ or Reelin^+^ cells were detected in controls (Figures 6A–A”,C–C”). However, the number of Prox1^+^ cells was increased approximately 5-fold in mutants (Figures 6B’,B”,G). Meanwhile, the number of Reelin^+^ CR cells in mutants was also increased approximately two-fold, but the increase was not as great as the increase in the number of Prox1^+^ cells (Figures 6D’,D”,G), consistent with our observations *in vivo*. Next, we detected the numbers of Foxp2^+^ cortical deep layer neurons and Satb2^+^ corpus callosal projection neurons. In controls, many Foxp2^+^ and Satb2^+^ cortical neurons did not co-express Prox1 (Figures 6E–E”’). However, the numbers of Foxp2^+^ and Satb2^+^ neurons were remarkably decreased in mutants (Figures 6F–F”’,G), suggesting that *Foxg1-*deficient cells did not develop into cortical neurons. This finding supports the hypothesis that FoxG1 plays a cell-autonomous role in repressing DG granule cell fate.

## Discussion

During the early development of the telencephalon, specific neuronal cell types are produced in a spatiotemporal manner and are organized into distinct functional regions. However, the mechanisms controlling cell fate determination remain unclear. In the present study, using temporal loss-of-function of *Foxg1*, we have revealed a cell-autonomous role for FoxG1 in repressing DG granule cell fate beginning at E10.5. Moreover, FoxG1 functions upstream of Lef1 to control cell fate, while the cortical hem itself might not significantly contribute to this process.

### Cell-Autonomous Role of FoxG1 in Repressing the DG Granule Cell Fate

Conventional KO of *Foxg1* (which is lost from E8.5 onward during forebrain development) causes the large-scale lateral-to-medial repatterning of the cortical primordium, and the neocortical plate is replaced by the expanded cortical hem and the medial pallium, which gives rise to the DG (Muzio and Mallamaci, 2005; Godbole et al., 2018). In the present study, when *Foxg1* was deleted at E10.5, the expansion of Prox1 expression followed a medial to lateral gradient, suggesting that the phenotype we observed might be a combination of repatterning and cell-autonomous effects. However, when *Foxg1* was deleted at E12.5, a time point when cortical patterning is complete (Shimogori et al., 2004; Storm et al., 2006; Borello and Pierani, 2010), we observed dramatic increases in the number of DG granule-like cells throughout the cortex. Our finding that the deletion of *Foxg1* at this time point only leads to a modest change in cortical hem markers indicates that, unlike the conventional KO, this phenotype is not likely to be caused by repatterning but rather a change in cell fate. Interestingly, when *Foxg1* was deleted at E14.5, we still observed extensive overproduction of granule-like cells in the cortex. In addition, mosaic deletion of *Foxg1* in the telencephalic neuroepithelium showed that *Foxg1-*deficient cells developed into granule-like cells, while adjacent normal progenitors developed into cortical neurons, strongly indicating a cell-autonomous role for FoxG1 in this process.

FoxG1 has been shown to regulate global gene expression (Kumamoto et al., 2013). It may serve as a potent regulator of DG granule cell fate by normally suppressing this fate in other cortical neurons. By directly repressing Wnt8b, FoxG1 inhibits Wnt/β -catenin signaling to control the development of the zebrafish forebrain. Here, we also detected altered Wnt signaling. Future studies will more directly examine how FoxG1 interacts with Wnt signaling pathway. A *Foxg1* deficiency in some cells at E9.5 results in a loss of Lhx2 expression, and these cells then form an ectopic hem, subsequently leading to the transformation of an adjacent area into a Prox1-, Lef1- and Ephb1-expressing DG-like region (Godbole et al., 2018). In this study, when *Foxg1* was deleted beginning at E10.5, no obvious changes in Lhx2 expression were detected, suggesting a spatiotemporal role for FoxG1 during the development of the telencephalon. Based on our data and previously published studies, prior to E10.5, FoxG1 is likely required for telencephalic patterning and cortical hem development (Hanashima et al., 2004; Muzio and Mallamaci, 2005; Hanashima et al., 2007; Manuel et al., 2009; Godbole et al., 2018), but from E10.5 onward, FoxG1 plays a cell-autonomous role that is crucial for repressing DG granule cell fate.

### FoxG1 Suppresses the Generation of CR Cells

When we disrupted *Foxg1* expression at E10.5 or E12.5, we observed significant increases in the CR cell number in the cortex. Our data on CR cells is consistent with previous studies; however, in contrast to these other studies, most cortical neurons did not adopt a CR cell fate but instead a greater proportion of *Foxg1*-deficient cells switched to a granule-like cell fate. Shen et al. (2006) have shown that knock down of *Foxg1* in cultured cortical progenitor cells increases the number of Reelin^+^ cells. Here, we cultured *Foxg1-*deficient cortical progenitor cells and observed an increase percentage of Reelin^+^ cells, although most of the progenitors adopted a granule-like cell fate instead. Based on these results, beginning at E10.5, FoxG1 represses both CR and granule cell fates. Additional studies are needed to elucidate the distinct mechanisms by which FoxG1 represses both CR cell and granule cell fates.

## Author Contributions

CZ, XH, and XG designed the research. CZ, XH, and SP wrote the paper. XH and XG performed immunostaining. XH and QZ performed the *in situ* hybridization. XH, XG, and CZ analyzed the data. XH performed the *in vitro* primary cell culture. QW performed the immunostaining of cultured cells. XH and YC performed the quantitative real-time PCR. QW performed the cell counting. XH performed the statistical analyses.

## Conflict of Interest Statement

The authors declare that the research was conducted in the absence of any commercial or financial relationships that could be construed as a potential conflict of interest.
